# Circular RNA in Chemonaive Lymph Node Negative Colon Cancer Patients

**DOI:** 10.3390/cancers13081903

**Published:** 2021-04-15

**Authors:** Inge van den Berg, Marcel Smid, Robert R. J. Coebergh van den Braak, Carolien H. M. van Deurzen, Vanja de Weerd, John A. Foekens, Jan N. M. IJzermans, John W. M. Martens, Saskia M. Wilting

**Affiliations:** 1 Department of Surgery, Erasmus MC-University Medical Center Rotterdam, 3015 GD Rotterdam, The Netherlands; i.vandenberg@erasmusmc.nl (I.v.d.B.); r.coeberghvdbraak@erasmusmc.nl (R.R.J.C.v.d.B.); j.ijzermans@erasmusmc.nl (J.N.M.I.); 2 Department of Medical Oncology, Erasmus MC Cancer Institute, University Medical Center Rotterdam, 3015 GD Rotterdam, The Netherlands; m.smid@erasmusmc.nl (M.S.); v.deweerd@erasmusmc.nl (V.d.W.); j.foekens@erasmusmc.nl (J.A.F.); j.martens@erasmusmc.nl (J.W.M.M.); 3 Department of Pathology, Erasmus MC-University Medical Center Rotterdam, 3015 GD Rotterdam, The Netherlands; c.h.m.vandeurzen@erasmusmc.nl

**Keywords:** colon cancer, circular RNA, prognostic stratification, CMS

## Abstract

**Simple Summary:**

Colon cancer (CC) is one of the most common types of cancer. Circular RNAs (circRNAs) appear to play an important role in tumor progression of CC. They are stably expressed in saliva, blood, and exosomes, potentially rendering them promising biomarkers for the diagnosis, prognosis, and treatment of CC. In this study we describe the identification of an extensive catalog of circRNAs in a large cohort of 181 chemonaive, stage I/II primary colon tumors and related circRNA expression to consensus molecular subtypes (CMS), microsatellite instability (MSI) status and clinical outcome. We observed that a high diversity in circRNAs was associated with favorable disease-free survival, and that several circRNAs were associated with MSI and CMS, demonstrating the potential clinical value of circRNAs in CC.

**Abstract:**

Circular RNAs (circRNAs) appear important in tumor progression of colon cancer (CC). We identified an extensive catalog of circRNAs in 181 chemonaive stage I/II colon tumors, who underwent curative surgery between 2007 and 2014. We identified circRNAs from RNAseq data, investigated common biology related to circRNA expression, and studied the association between circRNAs and relapse status, tumor stage, consensus molecular subtypes (CMS), tumor localization and microsatellite instability (MSI). We identified 2606 unique circRNAs. 277 circRNAs (derived from 260 genes) were repeatedly occurring in at least 20 patients of which 153 showed a poor or even negative (*R* < 0.3) correlation with the expression level of their linear gene. The circular junctions for circSATB2, circFGD6, circKMT2C and circPLEKHM3 were validated by Sanger sequencing. Multiple correspondence analysis showed that circRNAs were often co-expressed and that high diversity in circRNAs was associated with favorable disease-free survival (DFS), which was confirmed by Cox regression analysis (Hazard Ratio (HR) 0.60, 95% CI 0.38–0.97, *p* = 0.036). Considering individual circRNAs, absence of circMGA was significantly associated with relapse, whereas circSATB2, circNAB1, and circCEP192 were associated with both MSI and CMS. This study represents a showcase of the potential clinical utility of circRNAs for prognostic stratification in patients with stage I–II colon cancer and demonstrated that high diversity in circRNAs is associated with favorable DFS.

## 1. Introduction

Colon cancer is one of the most common types of cancer with over 1 million new cases worldwide and around 9800 new cases in the Netherlands in 2018 [1]. Up to 21% of patients with stage I–II colon cancer and 40% of patients with stage III colon cancer will develop metastatic disease after curative surgery [2]. As much of the disparity in prognosis for clinically comparable patients remains unexplained, efforts are being directed at finding so far unknown factors that may play a role in the development and progression of colon cancer.

Transcriptome sequencing studies have identified many short and long RNAs with non-protein-coding ability [3,4,5,6]. These non-coding RNAs (ncRNAs) have received increasing attention in recent years due to their aberrant expression features associated with colorectal cancer (CRC) carcinogenesis [7,8,9]. Recent studies have shown that non-coding microRNAs can function as promising biomarkers for stage II [10,11] and stage I–II colon cancer patients [12]. Circular RNAs (circRNAs) represent a re-discovered, abundant class of non-coding RNA molecules [13]. Altered expression of circRNAs is observed in cancer tissue compared to normal tissue [14,15,16,17,18,19], and particularly in CRC [20]. circRNA biogenesis derives from back-splicing, but the regulation and the frequency of this event are under investigation [20]. circRNAs form covalently closed, continuous loop structures produced through an end-to-end formation during transcription [21,22,23]. Increasing evidence shows that circRNAs can function as miRNA sponges, transcription regulators, and interfere with splicing, as well [20]. They are conserved, abundant and often exhibit tissue-, developmental-, and stage-specific expression [24,25].

Due to their special circular structure, circRNAs are usually more stable than linear RNAs and are not easily degraded by exonucleases. They have been proven to remain stable in saliva, blood, and exosomes, which makes them promising biomarkers for the diagnosis, prognosis, and therapeutic assessment of cancer patients [26]. Recently, two novel circRNAs, both derived from the gene BCL2L12, were identified as biomarkers for stage II CRC patients [27]. Compared to conventional available cancer biomarkers (e.g., PSA and CEA), circRNAs are expected to have higher sensitivity and specificity in diagnosis and prognosis [28].

Taken together, these characteristics indicate that circRNAs could represent new clinical diagnostic and prognostic markers, and possibly provide new leads for the treatment of diseases. In this study we describe the identification of an extensive catalog of circRNAs in a large cohort of 181 chemonaive, stage I/II primary colon tumors and related these to tumor stage, localization, Consensus Molecular subtypes (CMS), microsatellite instability (MSI) status and clinical outcome.

## 2. Materials and Methods

### 2.1. Study Population and Patient Selection

Fresh-frozen tumor tissue was collected from 181 patients with stage I–II colon cancer undergoing curative surgery. These patients had been enrolled in the MATCH-study—a prospective multicenter cohort study in seven hospitals in the region of Rotterdam, the Netherlands-between 2007 and 2014. Patients have given informed consent on the storage and use of tissue samples, and the collection of clinical data for research purposes. The MATCH study was approved by the Erasmus MC IRB (MEC-2007-088). Inclusion criteria and additional clinical characteristics have been described [29]. 

Disease-free survival (DFS) was defined as the time elapsed between the date of surgery and either the date of any recurrence of disease or the date of the last follow-up visit at which a patient was considered to have no recurrence.

### 2.2. Sample Collection and Processing

Sample collection and processing, as well as RNA isolation and RNA sequencing have been described in detail previously [30,31,32]. All samples were reviewed by a pathologist (CHMvD) to ensure the presence of sufficient tumor cells (≥40%). Only samples with an RNA integrity number of at least 7.0 were selected for RNAseq analysis. RNA integrity numbers were assessed using the MultiNA Microchip Electrophoresis system (Shimadzu, Kyoto, Japan) [33].

#### 2.2.1. Microsatellite Instability

MSI analyses have previously been performed and described [32]. In short, the MSI analyses made use of the MSI Analysis System from Promega©, which is a fluorescent PCR-based assay for detection of microsatellite instability in seven markers, including five mononucleotide repeat markers (BAT-25, BAT-26, NR-21, NR-24 and MONO-27) and two pentanucleotide repeat markers (Penta C and Penta D) [34].

#### 2.2.2. Consensus Molecular Subtypes

The CMS classification was performed using the “CMSclassifier” package (https://github.com/Sage-Bionetworks/CMSclassifier, accessed on 23 May 2019), using the single-sample prediction parameter [35].

#### 2.2.3. Identification of circRNAs

The methodology used to identify circRNA reads has previously been described in detail [36]. In short, the method developed by Smid et al. uses sequence reads that have a “secondary alignment” (SA) tag. When using paired-end sequence data, and assuming a circRNA molecule is present, the sequence read that aligns over the crossing of the junction would “point toward” its read-mate somewhere in the circle. Aligning these reads to the linear reference, the junction read will get an SA tag which will be assigned to two locations if and only if this is the one and unique alignment configuration the STAR software can find [37,38]. The read-mate aligns somewhere in between these two locations. Finding additional read pairs showing this configuration with a breakpoint at the exact same location strengthens the evidence for circular transcripts. We included only regions with at least five reads crossing the circular junction. After filtering, GENCODE annotation was used to obtain the exon locations of genes that exactly matched to the circular region. For each sample, STAR also gives the raw read counts for all genes. These were normalized (Trimmed Mean of M-values implemented in edgeR [39]), and the normalized read counts were used to correlate with the number of junction reads of the circular transcripts. The script is also available at https://bitbucket.org/snippets/MSmid/Le949d/identify-circularrna-reads (accessed on 30 October 2018).

#### 2.2.4. Multiple Correspondence Analysis (MCA)

For a substantial number of genes, only a linear transcript is detected in the majority of samples, which results in many missing values per circRNA. This in turn, complicates the use of standard cluster analysis for the identification of sample groups with similar circRNA-related biology. Therefore, circRNA data were considered categorical, i.e., a circRNA was scored as either “present” or “absent” in a sample. These categorical data are suitable for a multiple correspondence analysis (MCA), which is a generalized principle component analysis. An MCA generates a combined plot that shows both patients and circRNAs in such a way that patients and circRNAs that have similar patterns are closer together. Thus, the colon cancer tumor samples and circRNAs are projected onto the same plane, in which the relative distance to either the samples or the circRNAs is meaningful. The 0,0 point corresponds to a sample or circRNA with an average profile. The R-package “ade4” was used to perform the MCA in R version 3.4.1. Custom functions to plot the MCA results are available upon request of the authors.

#### 2.2.5. Reverse Transcription, Quantitative PCR, and Sanger Sequencing

Candidate circular RNAs were selected, and divergent primers were designed that are only able to amplify and detect the circular and not the corresponding linear mRNA (Table S1). Total RNA, isolated with RNA-Bee according to the manufacturer’s instructions (CS105B, TEL TEST), was reverse transcribed into cDNA with the H-minus RevertAid First Strand cDNA Synthesis Kit (K1632, ThermoFisher Scientific, Waltham, MA, USA), followed by an RNAse-H step (AM2293; Ambion). For Sanger sequencing, cDNA from five individual patient samples were used for PCR. cDNA was amplified for 35 cycles using Phusion high-fidelity DNA polymerase (ThermoFisher Scientific) in a total reaction volume of 25 μL, containing 400 nM of each primer and 160 µM dNTPs. For every circRNA two resulting PCR amplicons were purified from gel using the Qiaquick Gel Extraction Kit from Qiagen (Hilden, Germany) according to the manufacturer’s protocol and subjected to Quick Shot Sanger Sequencing by BaseClear BV (Leiden, The Netherlands).

### 2.3. Statistical Analyses

As indicated above for a substantial number of genes, only a linear transcript was detected in the majority of samples, which results in many missing values per circRNA. This hampers statistical analyses of circRNA expression levels and therefore we categorized the circRNA data into “present” or “absent” for statistical evaluation as well. We used circRNAs present in at least 20 samples to ensure a sufficient number of events for subsequent statistical analyses. STATA version 14 and SPSS Version 24.0 (SPSS, Inc., Chicago, IL, USA) were used to perform the statistical tests that are also indicated in the text. Cox’s proportional-hazards regression was used to evaluate the (log-transformed) number of uniquely present circRNAs per sample, hereafter called circRNA diversity, with DFS, or as “present”/“absent” when evaluating individual circRNAs. Survival curves were evaluated using the logrank test (for individual circRNAs) or with the logrank test for trend (for circRNA diversity, after dividing into three equal quantiles). Pearson’s correlation was used to correlate the circRNA expression with the expression of the linear gene it was derived from. Analyses between categorical variables (like present/absent of a circRNA versus MSI yes/no) were analyzed using Fisher’s exact test. Reported *p*-values are two-sided and considered significant at *p* ≤ 0.05. *p*-values were corrected for multiple testing using Benjamini–Hochberg’s FDR correction when evaluating multiple circRNAs, which were considered significant at *p* < 0.10. 

## 3. Results

### 3.1. CircRNA Expression in Colon Cancer

We analyzed RNAseq data of 181 patients with chemonaive, stage I/II primary colon cancer. The median follow-up time was 53 months (IQR 37–59). Clinical and histopathological characteristics are listed in Table 1.

**Figure 1:**
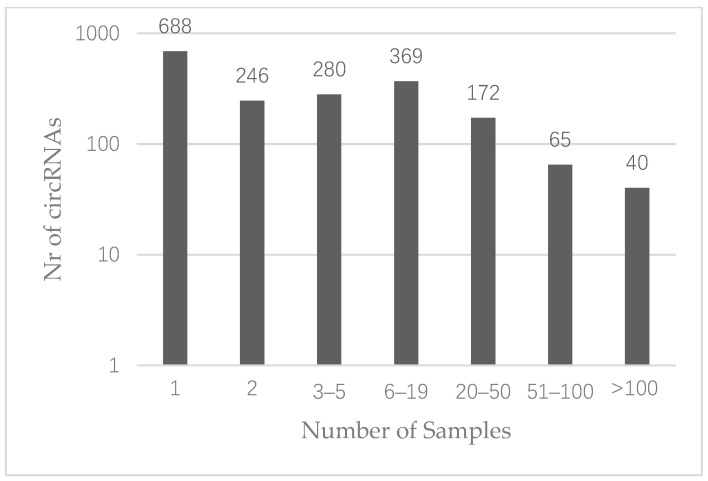
Histogram showing the distribution of circRNA occurrence in the 181 stage I/II colon cancer samples. A total of 1860 distinct circRNAs were identified which were derived from known genes.

Circular RNAs were defined as present when at least five reads crossed the circular junction [36]. This resulted in the identification of 2606 distinct circRNAs in the entire cohort, of which 1860 were derived from known genes. Sixty-three percent of these were repeatedly occurring in at least two colon cancer samples (*n* = 1172) (Figure 1), whereas 277 (15%) were observed in 20 samples or more (Table S2). The most repeatedly occurring circRNAs were derived from SMARCA5, HIPK3, ZKSCAN1 and FBXW7 and were observed in 177 samples each (n.b. not the same 177 samples for all four circRNAs) (Table 2). For 29 genes we observed that more than one unique circRNA was derived from the linear sequence (Table S2). Relative to the total number of samples expressing at least one circRNA from the respective gene, varying levels of co-expression between circRNAs derived from the same gene were observed with a median of 26.19% (range: 4.40–82.30%).

**Figure 2:**
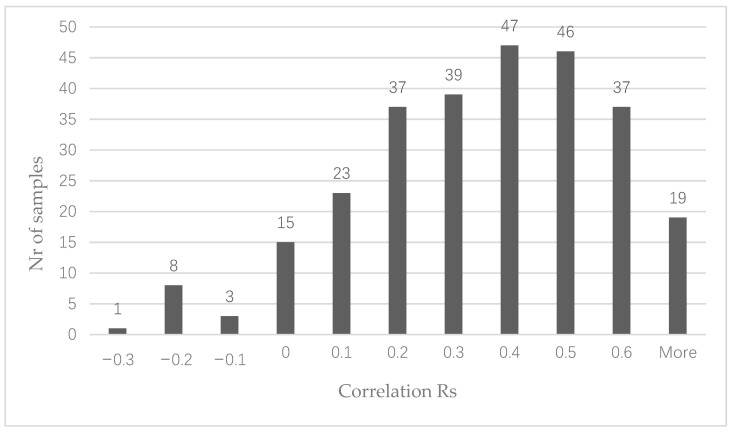
Histogram showing the distribution of the observed correlations between the number of circRNA and mRNA reads per gene.

We correlated the number of circRNA reads per circRNA with the expression of the linear gene from which the circRNA was derived. To avoid possible spurious correlations, only the 277 circRNAs found in at least 20 samples were analyzed. The vast majority of circRNAs showed a positive correlation with the linear gene from which the circRNA is derived (Figure 2). However, this correlation was poor (*R* < 0.3) for 126 circRNAs. Twenty-seven circRNAs showed a negative correlation with their corresponding linear gene, suggesting the circRNA may function independently from the linear transcript.

**Figure 3:**
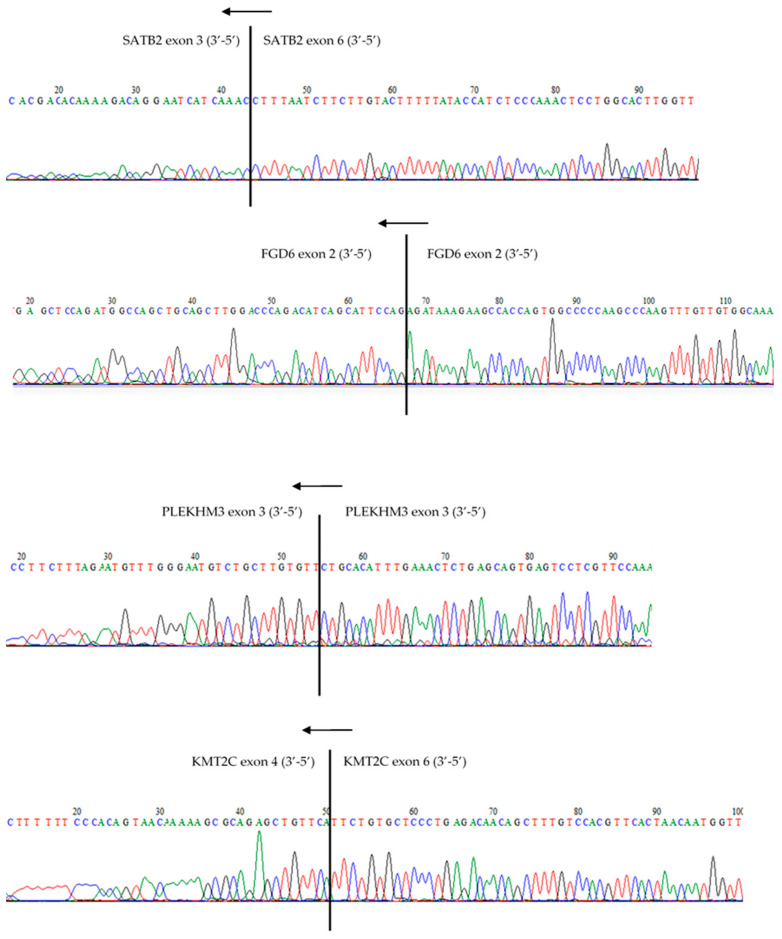
Sanger sequencing was used to validate the circular exon junctions of circSATB2, circKMT2C, circFGD6, and circPLEKHM3 identified from the RNAseq data.

We randomly selected four circRNAs representative of the entire list of identified circRNAs to get an unbiased validation of our circRNA identification pipeline. Our four candidates include two from the top-20 circRNAs showing the most positive and negative correlations to their linear counterparts respectively (SATB2_chr2:199368605-199433515, *R* = 0.61 and PLEKHM3_chr2:207976651-207977587, *R* = −0.17), and one novel circRNA (FGD6 (chr12:95208843-95211268), *R* = 0.39) not present in circBase (circbase.org (accessed on 23 May 2019) [40]). The identified junctions in the RNAseq data were verified for all four selected circRNAs by Sanger sequencing, thereby demonstrating the validity of our circRNA identification algorithm [36] (Figure 3).

### 3.2. CircRNA Expression Patterns Are Associated with Relevant Clinical Factors

For a substantial number of genes, only a linear transcript is detected in the majority of samples, which results in many missing values per circRNA. This in turn complicates the use of standard cluster analysis for the identification of circRNA/sample groups with similar circRNA-related biology. To be able to investigate circRNA profiles, we categorized circRNAs as “present” or “absent” in a sample and used this in a multiple correspondence analysis (MCA) to find naturally occurring subgroups. An MCA plot projects the colon tumor samples and circRNAs onto the same plane, in which the relative distance to either the samples or the circRNAs is meaningful. As such, samples that group close together have more similar circRNA profiles. In addition, since circRNAs have two states (present/absent), both these states are used in the analysis. Thus, two circRNAs that are “present” frequently in the same samples (co-occurrence) will be placed at a short distance, but this is also true for circRNAs that are mutually exclusive (presence of a circRNA and absence of the other circRNA) across the samples. Coloring the circRNA states will reveal the co-occurrence/mutual-exclusivity.

**Figure 4:**
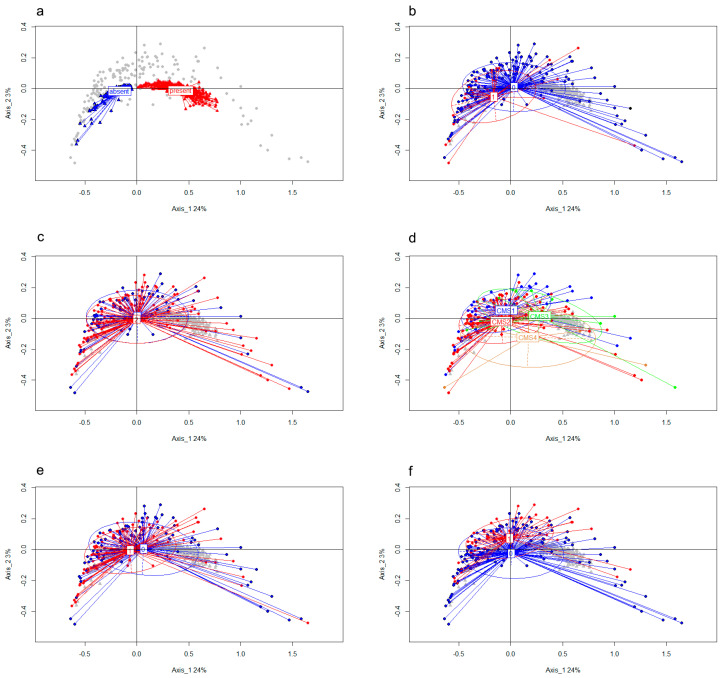
MCA analysis plots in which samples (closed circles) and circRNAs (triangles) are projected onto the same plane. (**a**). Blue and red indicate circRNAs without (absent) or with (present) circRNA expression, where similarity of two circRNA expression profiles (either both present (both red), both absent (both blue), or mutually exclusive (one red and one blue)) over the samples results in a small relative distance between these circRNAs. (**b**). Samples are colored based on relapse status, red indicates patients who relapse (1) showing circRNA profiles that are close to the “circRNA absent” group. (**c**). Samples are colored based on tumor stage I (blue) or stage II (red) showing no clear distinction in circRNA profiles. (**d**). The consensus molecular subtype (CMS) of the samples are indicated. CMS3 samples are most closely located to the “circRNA present” group. (**e**). Samples colored based on tumor side (left = 0; in blue, right = 1; in red) and (**f**), based on microsatellite instability (MSI) (MSI = 1; in red, MSS = 0; in blue).

For the MCA analysis, we used the 277 repeatedly occurring circRNAs (i.e., those which are present in at least 20 samples) and labelled these in each sample as “present” or “absent” as defined above. After MCA analysis, we first colored genes based on the circRNA state (Figure 4a). As shown by the clear separation of the “present” and “absent” state, circRNAs do not show mutual-exclusivity (which would show up as a red triangle among blue triangles or vice versa), but rather are often co-expressed in the same samples. Furthermore, the variability (spread among the *x*-axis) of the “present” profiles indicates different circRNA expression profiles are present among the samples, or, in other words, samples show a large diversity of expressed circRNAs.

**Figure 5:**
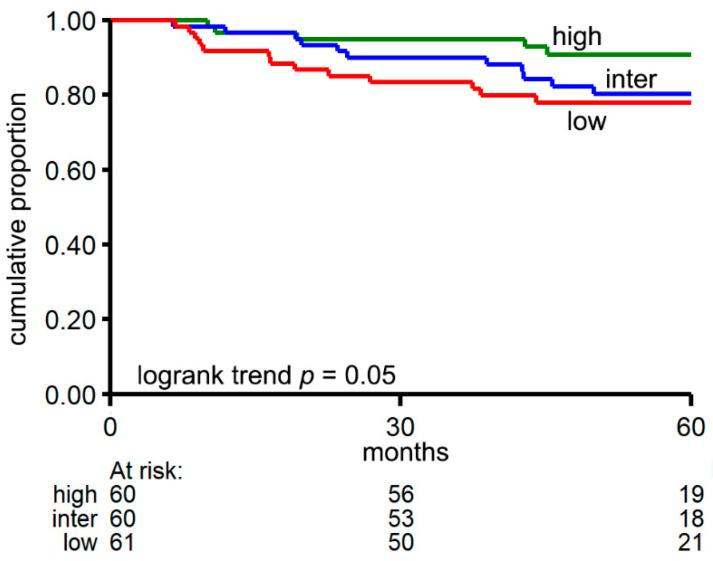
Kaplan–Meier survival curves of disease free survival in which patients were grouped in 3 equal quantiles based on their diversity in circRNA expression. The red line represents the quantile with the lowest diversity in circRNAs, the blue line represents the quantile with intermediate diversity in circRNAs and the green line represents the quantile with the highest diversity in circRNAs.

Next, we colored samples according to relapse status (Figure 4b), tumor stage (Figure 4c), CMS (Figure 4d), tumor localization (Figure 4e) and MSI (Figure 4f). Patients showing relapse (1) (Figure 4b) have profiles that are close to the “absent” group (group without circRNAs), which indicates that few genes give rise to circRNA expression in these samples. Indeed, when analyzing circRNA diversity (the number of distinct circRNA molecules in a sample) we found that a high diversity in circRNAs is associated with a favorable DFS: Cox regression using circRNA diversity as (log-transformed) continuous variable: Hazard Ratio (HR) 0.60, 95% CI 0.38–0.97, *p* = 0.036. Figure 5 shows Kaplan–Meier curves in which the levels of diversity of circRNAs were split into three equal quantiles to visualize the association between circRNA-diversity and DFS. The difference in DFS between the three quantiles was evaluated using the logrank test for trend, to account for the ordered structure of the sample groups (high, intermediate and low circRNA diversity; *p* = 0.050). High diversity was not associated with other clinical factors such as tumor stage, tumor side, MSI status, or CMS (diversity as continuous variable). 

The MCA plot of tumor side (Figure 4e) shows that right-sided tumors are closer to the absent group (group of samples without circRNAs) than the left-sided tumors –therefore also closer to the relapse group, but this association was not significant. Combining CMS grouping (Figure 4d) and MSI (Figure 4f) shows that, as expected, samples that are CMS1 and those that are MSI tumors have a similar position. Combining CMS (Figure 4d) and circRNA diversity (Figure 4a) leads to the conclusion that CMS3 patients have the highest diversity in circRNAs, and CMS2 patients the lowest. As to tumor stage, there was no clear distinction between stage I and II tumors with regard to circRNAs (Figure 4e). 

**Figure 6:**
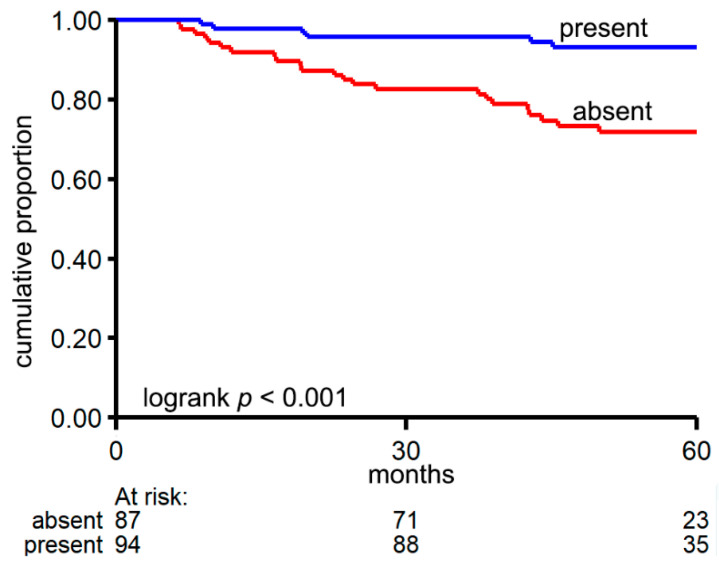
Kaplan–Meier analysis of patients in whom circMGA was detected (*n* = 94) versus patients in whom circMGA was not detected (*n* = 87).

Next to this global analysis of overall circRNA profiles in the samples, we also investigated whether the presence/absence of specific circRNAs was associated with relapse status, tumor stage, CMS, tumor localization, and MSI. Whereas no specific circRNAs were significantly associated with tumor stage and localization, the presence/absence of nine circRNAs was associated with CMS and five with MSI (Table 3; Fisher exact test *p* < 0.0003; Benjamini-Hochberg corrected *p*-value < 0.1). circSATB2 (Special AT-rich sequence-binding protein 2), circNAP1 (Nucleosome assembly protein) and circCEP192 (Centrosomal Protein 192) each correlated with both MSI and CMS. Only absence of circMGA (MAX dimerization protein) was significantly associated with relapse (Table 3; Fisher exact test *p* = 0.0002; Benjamini–Hochberg corrected *p*-value = 0.06). Kaplan-Meier analysis showed that patients in whom circMGA was detected (*n* = 94) had a favorable DFS compared to patients in which circMGA was not detected (*n* = 87; log-rank test *p* < 0.001, Cox HR 0.22 95%CI 0.09–0.53, *p* < 0.001) (Figure 6).

## 4. Discussion

With the use of RNAseq data, we could establish the presence of a wide variety of circRNAs in chemonaive lymph node negative, stage I/II primary colon tumors. Previous studies have been limited by the small number of circRNAs screened, the small sample size and retrospective data. Our study, however, concerned 181 patients included in a prospective, multicenter cohort study, and is therefore, to our knowledge, the largest circRNA-based biomarker discovery study done in stage I/II colon cancer. 

The four most repeatedly occurring circRNAs that we found (177/181 samples), circSMARCA5, circHIPK3, circFBXW7 and circZKSCAN1, have also been described as such in previous studies. circSMARCA5 was reported to be induced during epithelial-to-mesenchymal transition, which is an important mechanism during the metastatic process that has been associated with the pathogenesis of several cancers [26,41,42,43,44]. circHIPK3 has been described to promote CRC growth and metastasis by sponging miR-7 [45]. Furthermore, previous research in CRC cell lines showed that circFBXW7 is conducive in controlling the progression of CRC through NEK2, mTOR, and PTEN signaling pathways [37]. The correspondence of our finding with previous results clearly underlines the validity of our approach in identifying circRNAs. In addition, we performed Sanger sequencing to verify four randomly selected circRNAs (circSATB2, circKMT2C circFGD6, and circPLEKHM3) and successfully validated the identified circular junctions for all four circRNAs.

In the studied cohort of chemonaive lymph node negative colon cancer patients, a first highlight was the finding that high diversity of circRNAs present in colon cancer tissue was associated with favorable DFS. Vo et al showed that across different cancer types, total circRNA abundance was lower in cancer compared to normal tissue, suggesting that the reduction of circRNA generation could be associated with loss of cellular differentiation [46]. More specifically, presence of circMGA was significantly associated with a favorable DFS. Together, these findings support the idea that circRNAs might play a functional role in cancer metastasis [26]. Recent studies provide evidence for a tumor suppressive role for the gene MGA (MAX dimerization protein) in colorectal cancer [47]. In lung adenocarcinoma, the molecular function of MGA appears to be antagonistic to that of MYC. To our knowledge, this is the first study associating the circRNA emanating from this gene with colon cancer or any other malignancies.

A second highlight of this study is the association between circRNAs and distinct colorectal cancer subtypes. Presence/absence of nine and five circRNAs was significantly associated with CMS and MSI, respectively, of which circSATB2, circNAB1, circCEP192 were overlapping. Although we were unable to find a suitable publicly available RNAseq dataset to validate the associations we found between circRNAs and clinical parameters in our cohort of stage I–II colon cancer patients, a number of the circRNAs we found to be associated with distinct subtypes of colon cancer were described before in cancer. circSATB2 has been described to play a notable role in the progression of lung cancer by binding to miR-326 [48], which in turn is associated with CRC [49]. The association between CEP192, NAB1 and CRC or other cancers, has not been described in previous studies. A role in CRC was proven for circZNF609 (Zinc Finger Protein 609), which is down-regulated in CRC tissue and promotes apoptosis in CRC by upregulating p53 [50]. circUBAP2 (ubiquitin associated protein 2) facilitates CRC progression by sponging miR-199a to upregulate VEGFA which implies that circUBAP2 may be a potential therapeutic biomarker for CRC [51]. Furthermore, circZBTB44 (Zinc Finger and BTB Domain Containing 44) and circZNF609 are both upregulated in acute lymphoblastic leukemia [52] of which especially circZNF609 has a known oncogenic potential in multiple other cancers as well [53,54,55,56,57]. CircASPH (Aspartate Beta-Hydroxylase) expression is upregulated in lung adenocarcinoma [58] and, finally, circFUT8 (Fucosyltransferase 8) functions as a tumor suppressor in bladder cancer cells where low circFUT8 was associated with poor prognosis, high histological grade, and lymph node metastasis [59]. The largest strength of this study is its prospective, multicenter study design and that it is, to our knowledge, the largest circRNA-based biomarker discovery study performed in stage I/II colon cancer. However, as mentioned before, a limitation of this study is that we were unable to find a suitable publicly available dataset to validate the associations we found between circRNAs and clinical parameters. Furthermore, some of the subgroup analyses, such as MSI, resulted in rather small sample sizes in outcome, increasing the chance of type II errors.

## 5. Conclusions

In conclusion, this study generated a comprehensive catalog of circRNAs in colon cancer and demonstrated the potential biological and clinical relevance of circRNAs in patients with stage I–II colon cancer. We demonstrated high diversity in circRNAs is associated with favorable DFS. As such, circRNAs represent a promising addition to the biomarker repertoire for colon cancer.

## Figures and Tables

**Table 1:** Clinical and histopathological characteristics.

Clinical Variables	Categories	*n* = 181	%
Gender	Female	92	(50.9)
	Male	89	(49.2)
Age (median, IQR)		70	(63–76)
Tumor stage	Stage I	66	(36.5)
	Stage II	115	(63.5)
T status	T2	66	(36.5)
	T3	110	(60.8)
	T4	5	(2.8)
Nodal status	N0 ≥ 10 nodes assessed	149	(82.2)
	N0 < 10 nodes assessed	32	(17.3)
Tumor grade	Good	16	(8.8)
	Poor	10	(5.5)
	Moderate	152	(84)
	Unknown	3	(1.7)
Location	Right	92	(50.8)
	Left	89	(49.2)
MSI status	MSI	44	(24.3)
	MSS	137	(75.7)
Relapse	No	152	(84)
	Yes	29	(16)

Abbreviations: MSI = Microsatellite instability, MSS = Microsatellite stable.

**Table 2:** Most frequently recurring circRNAs.

Circular Region	Ensembl Gene ID	Gene	Exons	Nr. of Samples	R *
chr4:143543509-143543973	ENSG00000153147	*SMARCA5*	15-16	177	0.296
chr11:33286413-33287512	ENSG00000110422	*HIPK3*	2	177	0.541
chr7:100023419-100024308	ENSG00000106261	*ZKSCAN1*	2-3	177	0.539
chr4:152411303-152412530	ENSG00000109670	*FBXW7*	2	177	0.437

* R indicates the Pearson correlation between the number of circRNA reads and mRNA reads for that gene.

**Table 3:** Association of presence/absence of circRNAs with relapse status, tumor stage, CMS and MSI.

Name	Ensemble Gene ID	Circular Region	Fisher *p*-Value	FDR *	Comparison
circSATB2	ENSG00000119042	chr2:199368605-199433515	2.47 × 10^−8^	6.84 × 10^−6^	MSI
			2.41 × 10^−8^	6.68 × 10^−6^	CMS
circNAB1	ENSG00000138386	chr2:190659158-190673153	6.48 × 10^−7^	0.000179	MSI
			0.00007584	0.02078	CMS
circZBTB44	ENSG00000196323	chr11:130260856-130261930	3.4 × 10^−5^	0.009347	MSI
circCEP192	ENSG00000101639	chr18:12999421-13019207	0.000152	0.041758	MSI
			0.00007808	0.021316	CMS
		chr18:12999421-13030609	0.000233	0.063043	CMS
circUBAP2	ENSG00000137073	chr9:33960826-33973238	0.000238	0.064846	MSI
circMGA	ENSG00000174197	chr15:41668828-41669959	0.000235	0.064979	DFSI
circASPH	ENSG00000198363	chr8:61618978-61653661	1.19 × 10^−7^	3.28 × 10^−5^	CMS
circCCSER2	ENSG00000107771	chr10:84438512-84477665	3.9 × 10^−5^	0.010728	CMS
circZNF609	ENSG00000180357	chr15:64499293-64500167	0.00017	0.046128	CMS
circFUT8	ENSG00000033170	chr14:65561337-65561767	0.00026	0.070135	CMS
circMRPS35	ENSG00000061794	chr12:27714780-27724187	0.000299	0.08035	CMS

* False discovery rate (Benjamini–Hochberg procedure). Abbreviations: MSI = microsatellite instability, CMS = consensus molecular subtype.

## Data Availability

The data that support the findings of this study are available on request from the corresponding author.

## Supplementary Materials

The following are available online at https://www.mdpi.com/article/10.3390/cancers13081903/s1, Table S1: Candidate circular RNA validation by Sanger Sequencing, Table S2: circRNAs that were observed in 20 samples or more.
