# Supplementary material for: Circular RNA in Chemonaive Lymph Node Negative Colon Cancer Patients
Source: Cancers (Basel). 2021 Apr 15;13(8):1903. doi: 10.3390/cancers13081903 (PMC8071322; doi:10.3390/cancers13081903)
Supplement: Supplementary file 1 [file cancers-13-01903-s001.zip › cancers-1166409-supplementary.pdf]

Supplementary Materials

Circular RNA in Chemonaive Lymph Node Negative Colon Cancer Patients

Inge van den Berg, Marcel Smid, Robert R.J. Coebergh van den Braak, Carolien H.M. van Deurzen, Vanja de Weerd, John A. Foekens, Jan N.M. IJzermans, John W.M. Martens and Saskia M. Wilting

Table S1. Candidate circular RNA validation by Sanger Sequencing.

Circular Region	Ensemble Gene ID	Gene	Oligonucleotide	Sequence (5'–3')	Tm (°C)	Amplicon Size (bp)
chr2:199368605-199433515	ENSG00000119042	*SATB2*	Forward	CAAACTCGGCGTGTTCTTCTC	59.9	148
			Reverse	AATGTGTCAGCAACCAAGTGC	58.3	
chr7:152309966-152315339	ENSG00000055609	*KMT2C*	Forward	ATCCAGGCGTTATTCTGAATTGT	59.9	147
			Reverse	TGCCAGATGGAAGAACCATTG	60.71	
chr12:95208843-95211268	ENSG00000180263	*FGD6*	Forward	ATTTGCCACAACAAACTTGGG	60.1	149
			Reverse	CAGCATGGAGGACGCTGAT	58.9	
chr2:207976651-207977587	ENSG00000178385	*PLEKHM3*	Forward	CTGCATAACAGCTTTGCCAGT	58.3	135
			Reverse	TGTCTTTGGAACGAGGACTCA	57.9	

Table S2. circRNAs that were observed in 20 samples or more.

Circular Region	Ensemble Gene ID	Gene	Exons	# Samples	R *	circID
chr4:143543509-143543973	ENSG00000153147	*SMARCA5*	15-16	177	0.295	hsa_circ_0001445
chr11:33286413-33287512	ENSG00000110422	*HIPK3*	2	177	0.541	hsa_circ_0000284
chr7:100023419-100024308	ENSG00000106261	*ZKSCAN1*	2-3	177	0.539	hsa_circ_0001727
chr4:152411303-152412530	ENSG00000109670	*FBXW7*	2	177	0.437	hsa_circ_0001451
chr1:117402186-117420650	ENSG00000198162	*MAN1A2*	2-5	172	0.489	hsa_circ_0000118
chr14:99458279-99465814	ENSG00000183576	*SETD3*	6-2	170	0.352	hsa_circ_0000567
chr5:123545417-123557565	ENSG00000151292	*CSNK1G3*	2-3	166	0.52	hsa_circ_0001522
chr14:32090502-32094387	ENSG00000100852	*ARHGAP5*	3-2	162	0.64	hsa_circ_0031583
chr19:23358430-23362726	ENSG00000167232	*ZNF91*	1-4	161	0.756	
chr14:32090502-32117288	ENSG00000100852	*ARHGAP5*	3	157	0.607	hsa_circ_0031584
chr5:168488602-168494651	ENSG00000113643	*RARS*	2-5	156	0.276	hsa_circ_0001550
chr20:32366384-32369124	ENSG00000171456	*ASXL1*	2-4	152	0.415	hsa_circ_0001136
chr10:7797047-7802855	ENSG00000165629	*ATP5C1*	3-8	151	0.341	hsa_circ_0007292
chr8:51860845-51861247	ENSG00000168300	*PCMTD1*	3	150	0.431	hsa_circ_0001801
chr5:137985257-137988316	ENSG00000031003	*FAM13B*	10-8	150	0.235	hsa_circ_0001535
chr2:61522611-61533904	ENSG00000082898	*XPO1*	4-2	147	0.194	hsa_circ_0001017
chr13:32517857-32527533	ENSG00000244754	*N4BP2L2*	5-2	144	0.463	hsa_circ_0000471
chr7:155672867-155680909	ENSG00000184863	*RBM33*	3-5	142	0.121	hsa_circ_0001772
chr6:47283938-47286596	ENSG00000146072	*TNFRSF21*	3-2	141	0.548	hsa_circ_0001610
chr8:61680968-61684189	ENSG00000198363	*ASPH*	3-2	139	0.525	hsa_circ_0084615
chr6:4891713-4892380	ENSG00000153046	*CDYL*	4	139	0.524	hsa_circ_0008285
chr7:22291175-22318038	ENSG00000136237	*RAPGEF5*	1-2	128	0.474	hsa_circ_0001681
chr17:20204333-20205913	ENSG00000128487	*SPECC1*	4	122	0.678	hsa_circ_0000745
chr15:58912563-58917000	ENSG00000137776	*SLTM*	5-3	119	0.203	hsa_circ_0000605
chr15:64499293-64500167	ENSG00000180357	*ZNF609*	2-1	117	0.368	hsa_circ_0000615
chr9:33971651-33973238	ENSG00000137073	*UBAP2*	8-7	116	0.156	hsa_circ_0001851
chr12:108652272-108654411	ENSG00000110880	*CORO1C*	8-7	115	0.212	hsa_circ_0000437
chr1:117402186-117442326	ENSG00000198162	*MAN1A2*	2-6	115	0.275	hsa_circ_0000119
chr12:69800209-69801722	ENSG00000127328	*RAB3IP*	7-8	114	0.293	hsa_circ_0000419
chr15:25405461-25411972	ENSG00000114062	*UBE3A*	4-2	112	0.356	hsa_circ_0000586
chr4:55411614-55417986	ENSG00000134851	*TMEM165*	2-4	112	0.339	hsa_circ_0001414
chr6:158312051-158314269	ENSG00000130338	*TULP4*	2-1	110	0.476	
chr4:128992167-129003877	ENSG00000151466	*SCLT1*	9-6	109	0.339	hsa_circ_0001439
chr20:35714185-35725156	ENSG00000131051	*RBM39*	10-6	109	0.407	hsa_circ_0004870
chr11:130260856-130261930	ENSG00000196323	*ZBTB44*	2	109	0.317	hsa_circ_0002484
chr2:40428473-40430305	ENSG00000183023	*SLC8A1*	2	107	0.61	hsa_circ_0000994
chr14:96833467-96860736	ENSG00000100749	*VRK1*	2-11	105	0.271	hsa_circ_0000566
chr3:149846011-149921228	ENSG00000082996	*RNF13*	2-8	102	0.512	hsa_circ_0001346
chr6:18236452-18258406	ENSG00000124795	*DEK*	9-3	102	0.446	hsa_circ_0075796
chr21:36247517-36248569	ENSG00000142197	*DOPEY2*	20-21	101	0.388	hsa_circ_0001187
chr4:186706563-186709846	ENSG00000083857	*FAT1*	2	96	0.339	hsa_circ_0001461
chr7:158759486-158764854	ENSG00000117868	*ESYT2*	13-9	94	0.398	hsa_circ_0001776
chr2:190659158-190673153	ENSG00000138386	*NAB1*	2-4	94	0.661	hsa_circ_0002024
chr15:41668828-41669959	ENSG00000174197	*MGA*	2	94	0.09	hsa_circ_0000591
chr1:7777160-7778170	ENSG00000049245	*VAMP3*	3-4	93	0.05	hsa_circ_0006354
chr9:33953285-33963792	ENSG00000137073	*UBAP2*	12-9	91	0.247	hsa_circ_0001847
chr2:199368605-199433515	ENSG00000119042	*SATB2*	6-3	90	0.607	hsa_circ_0003915
chr3:56592970-56594029	ENSG00000180376	*CCDC66*	8-9	90	0.295	hsa_circ_0001313
chr9:135881633-135883079	ENSG00000130559	*CAMSAP1*	3-2	90	0.197	hsa_circ_0001900
chr10:126970702-127127765	ENSG00000150760	*DOCK1*	2-27	89	0.504	hsa_circ_0020397
chr9:93471141-93498887	ENSG00000048828	*FAM120A*	2	89	0.403	hsa_circ_0001875
chr14:22909483-22911404	ENSG00000100461	*RBM23*	3-2	88	0.154	hsa_circ_0000524
chr8:141253989-141254630	ENSG00000022567	*SLC45A4*	1-2	87	0.05	hsa_circ_0001829
chr7:22308339-22318038	ENSG00000136237	*RAPGEF5*	5-2	86	0.284	
chr1:31915895-31919659	ENSG00000184007	*PTP4A2*	2-2	86	0.439	hsa_circ_0007364
chr3:158122103-158123992	ENSG00000174891	*RSRC1*	2-3	85	0.295	hsa_circ_0001355
chr1:205616478-205623892	ENSG00000158711	*ELK4*	5-2	84	0.405	hsa_circ_0000175
chr17:45475100-45475727	ENSG00000225190	*PLEKHM1*	4	84	0.383	hsa_circ_0044177
chr7:39987599-40002032	ENSG00000065883	*CDK13*	2-5	84	0.481	hsa_circ_0001699
chr21:15762891-15766142	ENSG00000155313	*USP25*	2-3	84	0.11	hsa_circ_0001178
chr4:128074460-128077963	ENSG00000138709	*LARP1B*	2-4	79	0.32	hsa_circ_0001438

chr13:60439688-60467380	ENSG00000083544	*TDRD3*	2-4	77	0.529	hsa_circ_0003441
chr17:1050050-1100736	ENSG00000159842	*ABR*	16-3	76	0.468	hsa_circ_0007919
chr4:87195324-87195691	ENSG00000145332	*KLHL8*	2	76	0.313	hsa_circ_0002538
chr1:1223244-1223969	ENSG00000078808	*SDF4*	4-3	76	0.224	hsa_circ_0000002
chr2:72718103-72733119	ENSG00000144036	*EXOC6B*	6-3	75	0.287	hsa_circ_0009043
chr9:111386377-111391825	ENSG00000136813	*KIAA0368*	31-28	75	−0.072	hsa_circ_0001882
chr8:18799295-18804899	ENSG00000156011	*PSD3*	8-5	73	0.463	hsa_circ_0004458
chr12:32598497-32611284	ENSG00000139132	*FGD4*	5-10	72	0.555	hsa_circ_0025843
chr9:110972073-110973559	ENSG00000198121	*LPAR1*	3-2	71	0.494	hsa_circ_0087960
chr18:12999421-13019207	ENSG00000101639	*CEP192*	2-9	70	0.564	hsa_circ_0000831
chr12:46229153-46243315	ENSG00000111371	*SLC38A1*	5-2	70	0.383	hsa_circ_0000396
chr4:37631385-37638505	ENSG00000181826	*RELL1*	6-4	70	0.381	hsa_circ_0001400
chr6:158282263-158314269	ENSG00000130338	*TULP4*	1-2	69	0.406	
chr5:95755396-95763621	ENSG00000164292	*RHOBTB3*	6-7	69	0.575	hsa_circ_0007444
chr3:170359699-170361430	ENSG00000136603	*SKIL*	2	67	0.543	hsa_circ_0067938
chr6:116689320-116692393	ENSG00000196911	*KPNA5*	3-5	66	0.31	
chr16:85633914-85634133	ENSG00000131149	*GSE1*	2	65	0.19	hsa_circ_0000722
chr5:73074742-73077494	ENSG00000157107	*FCHO2*	20-21	65	0.421	hsa_circ_0002490
chr2:61522611-61526522	ENSG00000082898	*XPO1*	4-3	65	0.235	hsa_circ_0001016
chr3:170136419-170149245	ENSG00000173889	*PHC3*	7-5	64	0.631	hsa_circ_0001359
chr21:29321221-29329694	ENSG00000156273	*BACH1*	2-4	63	0.404	hsa_circ_0001181
chr14:65561337-65561767	ENSG00000033170	*FUT8*	3	63	0.519	hsa_circ_0003028
chr12:120154970-120155720	ENSG00000089154	*GCN1L1*	31-29	63	0.144	hsa_circ_0000448
chr12:27714780-27724187	ENSG00000061794	*MRPS35*	2-5	62	0.453	hsa_circ_0000384
chr14:21503173-21503882	ENSG00000165819	*METTL3*	2	61	−0.012	hsa_circ_0000523
chr15:89113725-89116522	ENSG00000140526	*ABHD2*	2-3	61	0.397	hsa_circ_0007099
chr20:58438945-58441084	ENSG00000124164	*VAPB*	4-5	61	0.436	hsa_circ_0001173
chr2:58221942-58232113	ENSG00000115392	*FANCL*	5-2	60	0.559	hsa_circ_0001009
chr1:23030469-23050521	ENSG00000004487	*KDM1A*	2-3	59	0.133	hsa_circ_0009061
chr18:12999421-13030609	ENSG00000101639	*CEP192*	2-11	58	0.509	
chr7:24623666-24668661	ENSG00000105926	*MPP6*	2-9	58	0.762	hsa_circ_0001686
chr1:155853276-155853807	ENSG00000116580	*GON4L*	2	58	−0.28	hsa_circ_0000139
chr8:130152736-130180881	ENSG00000153317	*ASAP1*	7-8	58	0.253	hsa_circ_0008934
chr7:129014979-129018158	ENSG00000064419	*TNPO3*	4-2	58	0.208	hsa_circ_0001741
chr9:86305192-86310018	ENSG00000083223	*ZCCHC6*	24-20	57	0.419	hsa_circ_0001869
chr4:185247294-185267156	ENSG00000109762	*SNX25*	2-5	57	0.618	hsa_circ_0004874
chr7:100812747-100813209	ENSG00000196411	*EPHB4*	12-11	55	0.342	hsa_circ_0001730
chr19:12928342-12928848	ENSG00000179115	*FARSA*	8-6	55	0.025	hsa_circ_0000896
chr2:112299849-112300030	ENSG00000188177	*ZC3H6*	2-2	55	0.556	hsa_circ_0001062
chr3:170145423-170149245	ENSG00000173889	*PHC3*	6-5	54	0.69	hsa_circ_0001360
chrX:131749306-131794467	ENSG00000213468	*FIRRE*	10-5	53	0.808	hsa_circ_0001944
chr9:37126312-37126943	ENSG00000147905	*ZCCHC7*	2	52	0.407	hsa_circ_0001860
chr3:63912588-63913226	ENSG00000163635	*ATXN7*	4	52	0.25	hsa_circ_0007761
chr1:224952670-224974154	ENSG00000185842	*DNAH14*	2-8	51	0.412	hsa_circ_0016600
chr1:41070595-41075452	ENSG00000010803	*SCMH1*	9-8	50	−0.025	hsa_circ_0000061
chr13:95757644-95763954	ENSG00000102580	*DNAJC3*	5-9	50	0.241	
chr1:155438327-155459899	ENSG00000116539	*ASH1L*	5-4	49	0.034	hsa_circ_0003247
chr4:76134175-76144474	ENSG00000138750	*NUP54*	4-2	49	0.199	hsa_circ_0070039
chr2:99169550-99171430	ENSG00000158411	*MITD1*	3-4	48	0.045	hsa_circ_0001050
chr8:140864312-140890770	ENSG00000169398	*PTK2*	5-3	48	0.031	hsa_circ_0002483
chr13:75560753-75569508	ENSG00000118939	*UCHL3*	3-6	48	0.353	hsa_circ_0000494
chr3:172247533-172251542	ENSG00000075420	*FNDC3B*	5-6	47	0.362	hsa_circ_0006156
chr10:101667886-101676437	ENSG00000107829	*FBXW4*	5-2	47	−0.051	hsa_circ_0008362
chr8:127890589-127890999	ENSG00000249859	*PVT1*	3-2	47	0.356	hsa_circ_0001821
chr15:62007308-62013993	ENSG00000129003	*VPS13C*	13-8	46	0.166	hsa_circ_0000607
chr10:110964125-110985766	ENSG00000108061	*SHOC2*	2-3	46	0.349	hsa_circ_0020028
chr16:69370483-69372356	ENSG00000132604	*TERF2*	5-4	46	−0.069	
chr3:125313308-125331239	ENSG00000163848	*ZNF148*	4-2	46	0.363	hsa_circ_0001333
chr14:39179091-39179463	ENSG00000100941	*PNN*	6-8	46	−0.02	
chr8:94664697-94665197	ENSG00000104413	*ESRP1*	7-9	46	0.291	hsa_circ_0084927
chr2:147896301-147899899	ENSG00000121989	*ACVR2A*	2-4	45	0.341	hsa_circ_0001073
chr19:8455405-8463687	ENSG00000099783	*HNRNPM*	2-5	45	−0.223	hsa_circ_0006382
chr7:152309966-152315339	ENSG00000055609	*KMT2C*	6-4	45	−0.033	hsa_circ_0001769
chr17:51263274-51268905	ENSG00000011260	*UTP18*	2-4	44	0.132	hsa_circ_0002789

chr14:102040236-102040674	ENSG00000197102	*DYNC1H1*	63-64	44	−0.212	hsa_circ_0002398
chr20:33619517-33623297	ENSG00000078699	*CBFA2T2*	4-5	44	0.264	hsa_circ_0003426
chr4:73090667-73092301	ENSG00000132466	*ANKRD17*	29	44	0.204	hsa_circ_0007883
chr7:23611171-23611554	ENSG00000169193	*CCDC126*	3	43	0.49	hsa_circ_0001684
chr5:57246300-57251142	ENSG00000062194	*GPBP1*	8-11	43	0.253	hsa_circ_0072547
chr7:158788004-158799073	ENSG00000117868	*ESYT2*	6-2	43	0.551	hsa_circ_0001777
chr18:21765772-21779686	ENSG00000101752	*MIB1*	2-6	42	0.513	hsa_circ_0000835
chr5:128138597-128152806	ENSG00000064651	*SLC12A2*	8-15	42	0.598	hsa_circ_0006034
chr7:156826605-156836886	ENSG00000105983	*LMBR1*	4-2	42	0.345	hsa_circ_0005939
chr3:138570318-138571357	ENSG00000114107	*CEP70*	6-4	41	0.539	hsa_circ_0002468
chr12:120782655-120784594	ENSG00000157837	*SPPL3*	6-4	41	−0.013	hsa_circ_0003472
chr12:123586747-123590450	ENSG00000086598	*TMED2*	2-3	41	0.265	hsa_circ_0000458
chr2:202464809-202467690	ENSG00000204217	*BMPR2*	2-3	41	0.183	hsa_circ_0003218
chr2:8908621-8958643	ENSG00000143797	*MBOAT2*	4-2	40	0.71	hsa_circ_0000972
chr2:88782734-88792495	ENSG00000230006	*ANKRD36BP2*	4-12	40	0.465	
chr4:128074460-128082306	ENSG00000138709	*LARP1B*	2-5	40	0.493	hsa_circ_0007619
chr5:145817894-145826201	ENSG00000186314	*PRELID2*	5-2	39	0.562	hsa_circ_0006528
chr1:117402186-117405646	ENSG00000198162	*MAN1A2*	2-3	39	0.444	hsa_circ_0000116
chr12:95208843-95211268	ENSG00000180263	*FGD6*	2	38	0.39	
chr2:233388257-233390484	ENSG00000077044	*DGKD*	2-3	38	−0.242	hsa_circ_0001112
chr8:60741259-60743098	ENSG00000171316	*CHD7*	2	38	0.253	hsa_circ_0084582
chr19:47264603-47264947	ENSG00000105321	*CCDC9*	6-7	38	0.375	hsa_circ_0000944
chr11:77624963-77625819	ENSG00000074201	*CLNS1A*	4-3	38	0.451	hsa_circ_0000343
chr12:100282943-100298176	ENSG00000136021	*SCYL2*	2-4	38	0.178	hsa_circ_0006258
chr15:90439332-90443479	ENSG00000140575	*IQGAP1*	6-9	37	0.532	hsa_circ_0000651
chr7:66127704-66134375	ENSG00000249319	*AC068533.7*	8-10	37		hsa_circ_0004604
chr4:177353308-177360678	ENSG00000109674	*NEIL3*	8-9	37	0.307	hsa_circ_0001460
chr10:68959806-68960250	ENSG00000165732	*DDX21*	2	37	0.266	hsa_circ_0008865
chr6:13579451-13584226	ENSG00000124523	*SIRT5*	2-3	36	0.098	hsa_circ_0007218
chr1:59339958-59378838	ENSG00000172456	*FGGY*	3-5	36	0.218	hsa_circ_0006633
chr19:5604583-5604937	ENSG00000130254	*SAFB2*	11-10	36	0.017	hsa_circ_0000880
chr2:189791790-189818181	ENSG00000064933	*PMS1*	2-5	36	0.133	hsa_circ_0001083
chr8:37765526-37766356	ENSG00000147471	*PROSC*	2-4	36	0.184	hsa_circ_0001788
chr2:134253095-134254645	ENSG00000152127	*MGAT5*	2-3	35	0.228	hsa_circ_0001068
chr14:21230319-21234230	ENSG00000092199	*HNRNPC*	4-2	35	0.165	hsa_circ_0003643
chr3:146121112-146124230	ENSG00000152952	*PLOD2*	3-2	35	0.409	
chr11:77619606-77625819	ENSG00000074201	*CLNS1A*	1-3	35	0.326	hsa_circ_0023694
chr5:128131067-128141982	ENSG00000064651	*SLC12A2*	5-10	35	0.242	hsa_circ_0073762
chr3:196391813-196403020	ENSG00000163960	*UBXN7*	5-3	35	0.214	hsa_circ_0001380
chr1:30992390-30995221	ENSG00000134644	*PUM1*	7-6	35	0.199	hsa_circ_0000043
chr15:32526813-32533369	ENSG00000223509	*RP11-632K20.7*	1-2	34	0.579	
chr1:805799-810171	ENSG00000230092	*RP11-206L10.8*	4-2	34	0.633	hsa_circ_0002333
chr6:7176655-7189323	ENSG00000124782	*RREB1*	2-6	34	−0.203	hsa_circ_0001573
chr4:102304317-102315831	ENSG00000138821	*SLC39A8*	6-3	33	0.451	hsa_circ_0002782
chr12:28225795-28259443	ENSG00000123106	*CCDC91*	2-4	33	0.451	hsa_circ_0000386
chr10:126970702-127257430	ENSG00000150760	*DOCK1*	2-29	33	0.514	hsa_circ_0020399
chr15:80120328-80122801	ENSG00000086666	*ZFAND6*	3-5	33	0.01	hsa_circ_0000643
chr5:65988635-65994865	ENSG00000112851	*ERBB2IP*	2-4	33	0.407	hsa_circ_0001492
chr1:35358925-35361790	ENSG00000146463	*ZMYM4*	3-5	33	0.172	hsa_circ_0011536
chr8:108449823-108455931	ENSG00000104412	*EMC2*	3-5	33	0.175	
chr2:214767482-214781510	ENSG00000138376	*BARD1*	6-4	33	0.548	hsa_circ_0001098
chr2:207976651-207977587	ENSG00000178385	*PLEKHM3*	3	33	−0.168	hsa_circ_0001095
chr16:47497399-47515602	ENSG00000102893	*PHKB*	3-7	32	0.118	hsa_circ_0000698
chr11:85996826-86031612	ENSG00000073921	*PICALM*	12-2	32	0.18	hsa_circ_0023923
chr20:35716740-35725156	ENSG00000131051	*RBM39*	9-6	32	0.479	hsa_circ_0001147
chr10:84438512-84477665	ENSG00000107771	*CCSER2*	6-9	32	0.532	hsa_circ_0018998
chr6:138943513-138944623	ENSG00000135597	*REPS1*	7-5	32	0.45	hsa_circ_0004368
chr4:3086939-3107424	ENSG00000197386	*HTT*	2-5	32	−0.057	hsa_circ_0001392
chr4:48369849-48383785	ENSG00000109171	*SLAIN2*	2-6	31	0.413	
chr11:73707420-73718719	ENSG00000175582	*RAB6A*	6-4	31	0.373	hsa_circ_0000339
chr11:61366045-61367999	ENSG00000149483	*TMEM138*	3-4	31	0.098	hsa_circ_0002058
chr3:138570318-138572933	ENSG00000114107	*CEP70*	6-3	30	0.382	hsa_circ_0004524
chr1:45640210-45642500	ENSG00000159592	*GPBP1L1*	7-6	30	0.149	hsa_circ_0008774
chr10:31908172-31910564	ENSG00000165322	*ARHGAP12*	1-2	30	0.536	hsa_circ_0000231

chr2:106158058-106166084	ENSG00000115652	*UXS1*	5-2	29	0.004	hsa_circ_0001060
chr2:201145378-201149836	ENSG00000003402	*CFLAR*	6-8	29	0.093	hsa_circ_0001092
chr5:145796442-145826201	ENSG00000186314	*PRELID2*	1-2	29	0.545	hsa_circ_0008647
chr3:71041328-71053774	ENSG00000114861	*FOXP1*	11-8	29	0.845	hsa_circ_0008234
chr11:32927157-32935436	ENSG00000060749	*QSER1*	2-4	29	0.38	hsa_circ_0021570
chr6:158580940-158589783	ENSG00000146433	*TMEM181*	3-6	29	0.348	hsa_circ_0001661
chr1:26942660-26943066	ENSG00000090273	*NUDC*	7-6	29	0.159	hsa_circ_0005087
chr11:18291442-18292977	ENSG00000110756	*HPS5*	15-14	29	0.152	hsa_circ_0000280
chr7:22266964-22318038	ENSG00000136237	*RAPGEF5*	2	29	0.337	hsa_circ_0079557
chr8:37877109-37877552	ENSG00000156675	*RAB11FIP1*	2	28	0.229	hsa_circ_0001789
chr1:155438327-155439069	ENSG00000116539	*ASH1L*	5	28	0.091	hsa_circ_0000137
chr20:35729312-35732136	ENSG00000131051	*RBM39*	5-3	28	0.341	hsa_circ_0008817
chr9:33960826-33973238	ENSG00000137073	*UBAP2*	10-7	28	0.435	hsa_circ_0001850
chr8:61618978-61653661	ENSG00000198363	*ASPH*	14-4	28	0.429	hsa_circ_0084606
chr15:41696075-41699160	ENSG00000174197	*MGA*	2-5	28	−0.244	hsa_circ_0000592
chr9:37424845-37426655	ENSG00000137106	*GRHPR*	2-4	27	0.26	hsa_circ_0001861
chr5:154033791-154034968	ENSG00000055147	*FAM114A2*	4-2	27	0.45	hsa_circ_0001546
chr10:100923975-100926020	ENSG00000119906	*FAM178A*	5-6	27	0.378	hsa_circ_0006654
chr8:102360071-102361627	ENSG00000104517	*UBR5*	5-2	26	0.07	hsa_circ_0001819
chr6:13632370-13644730	ENSG00000010017	*RANBP9*	12-6	26	0.371	hsa_circ_0001577
chr6:30650994-30651467	ENSG00000204564	*C6orf136*	3-4	26	0.102	hsa_circ_0006109
chr1:20757166-20773611	ENSG00000127483	*HP1BP3*	9-5	26	−0.079	hsa_circ_0000024
chr14:63998914-64022864	ENSG00000054654	*SYNE2*	27-38	26	0.099	
chr9:125337018-125337592	ENSG00000165219	*GAPVD1*	14-15	26	0.152	hsa_circ_0003270
chr10:104008177-104018909	ENSG00000065613	*SLK*	12-14	26	0.366	hsa_circ_0000259
chrX:85303406-85308217	ENSG00000124429	*POF1B*	15-10	26	0.321	hsa_circ_0091187
chr1:70292388-70315567	ENSG00000118454	*ANKRD13C*	9-4	26	0.355	hsa_circ_0000085
chr4:105424196-105456746	ENSG00000138777	*PPA2*	7-2	26	0.431	hsa_circ_0001434
chr19:29985223-29986418	ENSG00000105176	*URI1*	3-4	25	0.116	hsa_circ_0000921
chr16:18841565-18845652	ENSG00000157106	*SMG1*	41-39	25	0.152	hsa_circ_0006434
chr7:131375424-131399434	ENSG00000128585	*MKLN1*	3-7	25	0.531	hsa_circ_0001746
chr5:36953618-36976403	ENSG00000164190	*NIPBL*	2-9	25	0.554	hsa_circ_0001472
chr9:96522506-96565484	ENSG00000081377	*CDC14B*	12-2	25	0.545	hsa_circ_0087641
chr7:92294889-92327901	ENSG00000001629	*ANKIB1*	2-5	25	0.332	
chr10:124681607-124682380	ENSG00000258539	*RP11-12J10.3*	9	25	−0.252	hsa_circ_0000267
chr2:230442937-230450256	ENSG00000067066	*SP100*	3-8	24	0.023	hsa_circ_0003922
chr5:176943335-176958155	ENSG00000087206	*UIMC1*	10-7	24	−0.07	hsa_circ_0001558
chr9:93471141-93476339	ENSG00000048828	*FAM120A*	2-3	24	0.419	hsa_circ_0008193
chr8:37870420-37877552	ENSG00000156675	*RAB11FIP1*	4-2	24	0.533	hsa_circ_0005630
chr10:84371014-84373816	ENSG00000107771	*CCSER2*	2-3	24	0.627	hsa_circ_0018992
chr20:41533050-41551361	ENSG00000124177	*CHD6*	3-2	24	0.218	hsa_circ_0001159
chr2:238182065-238185288	ENSG00000132323	*ILKAP*	9-6	24	0.278	hsa_circ_0001116
chr7:158869855-158876692	ENSG00000126870	*WDR60*	2-4	24	0.29	hsa_circ_0001778
chr1:112653598-112659780	ENSG00000116489	*CAPZA1*	4-7	24	0.144	hsa_circ_0000109
chr16:88027483-88038012	ENSG00000172530	*BANP*	7-10	23	0.052	hsa_circ_0040823
chr19:5047476-5082505	ENSG00000127663	*KDM4B*	6-9	23	0.055	hsa_circ_0002926
chr1:224952670-225007545	ENSG00000185842	*DNAH14*	2-8	23	0.637	hsa_circ_0016601
chr7:27629371-27649634	ENSG00000106049	*HIBADH*	4-2	23	0.194	hsa_circ_0006773
chr2:199380364-199433515	ENSG00000119042	*SATB2*	5-3	23	0.322	hsa_circ_0002867
chr19:40583398-40583718	ENSG00000160410	*SHKBP1*	11-12	23	−0.259	hsa_circ_0000936
chr1:29154696-29154911	ENSG00000116350	*SRSF4*	4	23	−0.062	hsa_circ_0006602
chr8:17743604-17755962	ENSG00000129422	*MTUS1*	2	23	0.424	hsa_circ_0083444
chr8:18765449-18804899	ENSG00000156011	*PSD3*	9-5	23	0.195	hsa_circ_0002111
chr1:20770930-20773611	ENSG00000127483	*HP1BP3*	6-5	23	−0.006	hsa_circ_0005782
chr1:8655973-8656442	ENSG00000142599	*RERE*	3	23	−0.428	
chr8:47396376-47407962	ENSG00000164808	*SPIDR*	6-7	22	0.017	hsa_circ_0001798
chr3:134188837-134195183	ENSG00000163785	*RYK*	9-7	22	0.102	hsa_circ_0005768
chr20:35721740-35725156	ENSG00000131051	*RBM39*	8-6	22	0.339	hsa_circ_0001148
chr4:90308244-90313048	ENSG00000184305	*CCSER1*	2-3	22	0.551	
chr14:39276934-39279538	ENSG00000258941	*RP11-407N17.3*	6-8	22		hsa_circ_0000530
chr18:9524594-9525852	ENSG00000017797	*RALBP1*	5-6	22	0.175	hsa_circ_0005158
chr17:67945409-67975959	ENSG00000171634	*BPTF*	22-27	22	0.636	hsa_circ_0000799
chr10:5794885-5800706	ENSG00000057608	*GDI2*	4-2	22	−0.015	hsa_circ_0002665
chr10:89751346-89762836	ENSG00000138182	*KIF20B*	24-29	22	0.563	hsa_circ_0019079

chr16:3850297-3851010	ENSG00000005339	*CREBBP*	2	21	−0.075	hsa_circ_0007637
chr7:131387120-131399434	ENSG00000128585	*MKLN1*	4-7	21	0.018	hsa_circ_0001747
chr6:110887505-110890357	ENSG00000123505	*AMD1*	2-4	21	0.277	hsa_circ_0005954
chr22:20933779-20934245	ENSG00000099942	*CRKL*	2	21	0.192	hsa_circ_0001206
chr8:98706467-98707312	ENSG00000104375	*STK3*	6-5	21	0.016	hsa_circ_0004592
chr20:51674153-51690820	ENSG00000054793	*ATP9A*	6-8	21	0.438	hsa_circ_0004770
chr3:56660731-56673726	ENSG00000163946	*FAM208A*	4-2	21	0.272	hsa_circ_0001315
chr19:34430576-34438767	ENSG00000126261	*UBA2*	3	21	0.367	hsa_circ_0006987
chr11:85996826-86003452	ENSG00000073921	*PICALM*	12-9	21	−0.143	hsa_circ_0023919
chr5:180261684-180280609	ENSG00000050748	*MAPK9*	5-2	21	0.463	hsa_circ_0001566
chr3:67495798-67508904	ENSG00000172340	*SUCLG2*	9-7	21	0.113	hsa_circ_0004276
chr4:87046166-87047595	ENSG00000172493	*AFF1*	3-4	21	−0.145	hsa_circ_0001423
chr17:59353215-59353527	ENSG00000175155	*YPEL2*	2	20	0.048	hsa_circ_0005600
chr13:112516440-112527485	ENSG00000126216	*TUBGCP3*	17-12	20	0.215	hsa_circ_0000504
chr4:51863437-51891852	ENSG00000109184	*DCUN1D4*	2-6	20	0.135	hsa_circ_0007646
chr5:16779545-16783470	ENSG00000145555	*MYO10*	9-5	20	0.2	
chr9:83678441-83686156	ENSG00000135018	*UBQLN1*	5-2	20	0.465	hsa_circ_0087357
chr8:70213903-70216765	ENSG00000140396	*NCOA2*	4-3	20	0.516	
chr13:75621763-75727099	ENSG00000261553	*RP11-29G8.3*	8-10	20	0.314	
chr2:88801099-88804881	ENSG00000230006	*ANKRD36BP2*	13	20	0.595	
chr16:69695136-69695380	ENSG00000102908	*NFAT5*	15	20	0.347	hsa_circ_0006845
chr7:139715932-139717016	ENSG00000064393	*HIPK2*	2	20	0.691	hsa_circ_0001756
chr7:43639449-43640650	ENSG00000106603	*COA1*	6-5	20	0.403	hsa_circ_0001700
chr10:34269657-34284246	ENSG00000148498	*PARD3*	22-21	20	0.345	hsa_circ_0018168

* R indicates the Pearson correlation between the number of circRNA reads and mRNA reads for that gene.

© 2021 by the authors. Licensee MDPI, Basel, Switzerland. This article is an open access article distributed under the terms and conditions of the Creative Commons Attribution (CC BY) license (http://creativecommons.org/licenses/by/4.0/).
